# Arch-Support Induced Changes in Foot-Ankle Coordination in Young Males with Flatfoot during Unplanned Gait Termination

**DOI:** 10.3390/jcm10235539

**Published:** 2021-11-26

**Authors:** Xuanzhen Cen, Lidong Gao, Meimei Yang, Minjun Liang, István Bíró, Yaodong Gu

**Affiliations:** 1 Faculty of Sports Science, Ningbo University, Ningbo 315211, China; 1811042008@nbu.edu.cn (X.C.); gaolidong1997@hotmail.com (L.G.); yangmeimei@nbu.edu.cn (M.Y.); liangminjun@nbu.edu.cn (M.L.); 2 Doctoral School on Safety and Security Sciences, Obuda University, 1034 Budapest, Hungary; 3 Faculty of Engineering, University of Szeged, 6720 Szeged, Hungary

**Keywords:** arch support, coupling angle, metatarsophalangeal joint, gait stop, vector coding technique

## Abstract

Objective: The efficacy of arch orthoses in posture adjustment and joint coordination improvement during steady-state gait is well documented; however, the biomechanical changes of gait sub-tasks caused by arch support (AS), especially during gait termination, are poorly understood. Hence, this study aimed to investigate how the acute arch-supporting intervention affects foot–ankle coordination and coordination variability (CV) in individuals with flatfoot during unplanned gait termination (UGT). Methods: Twenty-five male patients with flatfoot were selected as subjects participated in this AS manipulation study. A motion capture system was used for the collection of the metatarsophalangeal joint (MPJ) and ankle kinematics during UGT. MPJ-Ankle coordination and CV were quantified using an optimized vector coding technique during the three sub-phases of UGT. A paired-sample *t*-test from the one-dimensional statistical parametric mapping of one-dimensional was applied to examine the data significance. Results: Significant differences for the joint kinematics between non-arch-support (NAS) and AS were exhibited only in the MPJ transverse plane during the middle and later periods of UGT (*p* = 0.04–0.026). Frontal plane MPJ-ankle coordination under AS during stimulus delay significantly decreased from 177.16 ± 27.41° to 157.75 ± 32.54° compared with under NAS (*p* = 0.026); however, the coordination pattern had not changed. Moreover, no significant difference was found in the coupling angle variability between NAS and AS in three planes during sub-phases of UGT (all *p* > 0.5). Conclusions: The detailed intrinsic characteristic of AS induced acute changes in lower extremity segment coordination in patients with mild flatfoot has been recorded. This dataset on foot-ankle coordination characteristics during UGT is essential for explaining foot function and injury prediction concerning AS manipulation. Further studies are expected to reflect lower limb inter-joint coordination during gait termination through the long-term effects of AS orthoses.

## 1. Introduction

As one of the representative characteristics that distinguish humans from other primates, the medial longitudinal arch supports body weight and buffers loads while performing movement [1]. The triangular truss structure formed by the talus, metatarsal, and plantar aponeurosis can be used further to explain the complete arch function [2,3]. Unlike the standing posture, the body and the foot are relatively unbalanced during foot movement due to the center of mass (COM) change. During gait tasks such as walking and running, the supporting function of the foot arch becomes more complicated [4]. Arch curvature generally depends on the mutual balance between the foot muscles [5]. Loss of function or contracture of the foot muscles attached to the arch might cause an overall gait imbalance and even lead to foot deformity [5].

Among them, flatfoot is a typical foot deformity characterized by the medial arch’s reduction or disappearance, accompanied by calcaneus valgus and talus sinking [6,7]. Many studies have disclosed that flatfoot deformity increases the patients’ likelihood of experiencing knee or hip/back pain symptoms, soft tissue injury, and arthritis [8,9,10,11], as well as a decreased lower-limb motor function [12,13]. At present, the standard and conservative medical intervention for symptomatic flatfoot is orthotic treatment, such as correcting altered biomechanical characteristics of lower limbs by foot orthoses [14,15,16]. For example, Jafarnezhadgero et al. [14] reported that arch orthoses could significantly reduce the ankle, knee, and hip moment during ambulating in flexible-flatfooted children. Nevertheless, some researchers [17,18] have noted that orthotics have no significant beneficial effect on gait patterns in patients with flatfoot.

Although the effects of arch orthoses on routine gait tasks have been widely reported [14,15,16,17,18,19], there has been little attention given to gait sub-tasks, especially gait termination. Gait termination occurs when the feet stop moving forward or backward at the space-time level, and when the body no longer moves horizontally [20,21]. A previous study [4] noted that compared to steady-state gait, the magnitude of load received by the arch during the gait termination further increases. At the same time, due to the change of the motion pattern, the distribution of plantar relative regional impulse is different [22]. When people perform unplanned gait termination (UGT) in the face of unknown stimuli, it heightens the urgency for the dynamic stability to be activated spontaneously, leading to an increased risk of foot injury [21]. In addition, the gait termination task is also executed as an effective tool in gait analysis, which is widely applied in the evaluation of motor function in patients with concussion, multiple sclerosis, or chronic ankle instability [23,24,25,26,27]., As a foot deformation that can worsen the patient’s dynamic balance, the biomechanical mechanism of flatfoot in the process of UGT is also worth exploring.

Coordination patterns can assess the timing and degree of relative movement of inter-segment of the human body, allowing researchers to understand further the collaborative mechanism of the motor system [28]. Meanwhile, coordination variability (CV) can play an essential role in controlling the motion strategies by quantifying the coordination patterns’ level of fluctuation and improving the adaptive capacity to movement disturbances and task constraints [28,29,30]. Coordination and CV during moving have also been associated with biological systems’ health status [31]. Previous research [28] demonstrated that the 4-month usage of arch support (AS) orthoses could significantly improve joint coordination between ankle, knee, and hip, in patients with flatfoot during walking. However, the coordination pattern between the metatarsophalangeal joint (MPJ) and ankle joint, which is the closest anatomically to the foot arch, has been ignored. Evaluation of MPJ-ankle coordination and CV in flatfoot patients with AS through a vector coding method may provide valuable information about motion control, which is undoubtedly precious in a clinical setting [32].

To the best of our current knowledge, although some researchers [28] have attempted to analyze the long-term impact of foot orthoses on the inter-joint coordination during steady-state gait, few studies have verified the biomechanical mechanism of acute arch-support (AS) manipulation intervention during gait sub-tasks represented by gait termination. The acute effect from arch manipulation can reflect the essential characteristic in foot biomechanics and alleviate the potential confounding effects caused by long-term intervention, such as weight changes [33]. Hence, given the research interest in arch manipulation, the present study intended to reveal the foot biomechanical characteristics during UGT, including joint kinematics, MPJ-ankle coordination, and CV in individuals with flatfoot from AS manipulation.

## 2. Materials and Methods

### 2.1. Participants

#### 2.1.1. Preliminary Recruitment

Before the commencement of the biomechanical assessment, a power analysis utilizing GPower 3.1 (Franz Faul, Kiel University, Kiel, Germany) revealed that for effect size (ES) d of 0.6 (a power (1-β err prob) of 0.80 with a significance level (α err prob) of 0.05), the sample size of at least 24 participants was needed [34]. Therefore, twenty-five potential male patients with flatfoot were initially recruited in this study. The inclusion criteria were that patients needed to have at least a level of mild flatfoot, as visually assessed by a physiotherapist according to the classification standard of the previous study [35]. To further reduce the error caused by the subjective assessment, a static foot arch morphology evaluation, and a dynamic foot arch morphology evaluation were successively implemented to determine the subject’s arch morphology. Each participant understood the process and the objective of the experiment and submitted written informed consent before participation. The Ethics Committee at Ningbo University approved the procedures.

#### 2.1.2. Static Arch Evaluation

All patients’ three-dimensional (3D) arch morphology data during static standing were measured using the Easy-Foot-Scan acquisition system (OrthoBaltic, Kaunas, Lithuania). They needed to stand barefoot while keeping the COM steady without body shaking during the foot scanning stage. The acquisition system’s resolution, smoothness, and hole filling were resized as 1.0 mm, 30 mm, and 100 mm, respectively [4]. The 2D image obtained by static foot morphological scanning was referred to for measuring the required foot structure parameters with Auto CAD 2014 (Autodesk, San Francisco, CA, USA). The static arch index (SAI) was calculated as the height of the vertical from the instep to surface divided by the length from the 1st metatarsal to the calcaneus (ball of the foot length) [4,36]. Regarding the criterion established in the previous study [37], if the patient’s SAI was more significant than 0.26, they would be considered a flatfoot patient; otherwise, this patient would be excluded.

#### 2.1.3. Dynamic Arch Evaluation

After being assessed, patients were asked to walk barefoot along a 20 m walkway with a self-selected speed for the dynamic foot arch morphology evaluation. The center of the walkway contained a 2 m Footscan^®^ pressure system (RSscan International, Olen, Belgium) to record the dynamic plantar pressure data during walking with a sampling frequency of 87 Hz. The plantar surface of each subject was automatically partitioned into three anatomical sections by a Footscan^®^ version 7.0 software (RsScan International, Olen, Belgium). Specifically, a plantar axis was obtained from the midpoint of the 2nd and 3rd metatarsals to the midpoint of the foot calcaneus. Perpendicular to this axis, the plantar surface was partitioned into three anatomical sections: hindfoot (Area A), midfoot (Area B), and forefoot (Area C). The dynamic arch index (DAI) was defined as the contact area ratio between Area B and the entire foot without a big toe and other toes. This index was calculated applying the equation: DAI = Area B/(Area A + Area B + Area C) [4,38,39]. Based on the results of DAI, flatfoot would be determined if the index is greater than or equal to 0.28 [38].

**Figure 1:**
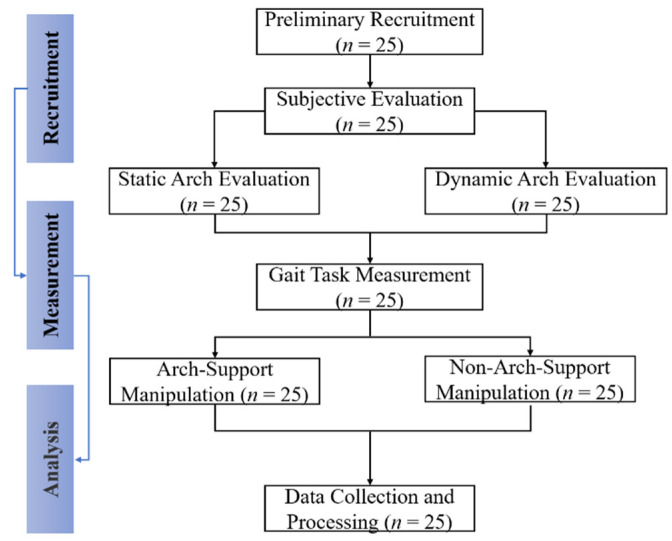
The flow chart of arch support manipulation intervention protocol.

After static and dynamic arch evaluations, a total of 25 participants were judged to be patients with flatfoot and were eventually included in gait task measurement (age: 25.80 ± 2.16 years, height: 175.68 ± 3.15 cm, weight: 70.52 ± 6.51 kg, BMI: 22.84 ± 1.90 kg/m^2^, foot length: 262.52 ± 2.33, SAI: 0.29 ± 0.02, DAI: 0.31 ± 0.02, the right-side leg is the dominant leg, Figure 1).

### 2.2. Gait Task Measurement

**Figure 2:**
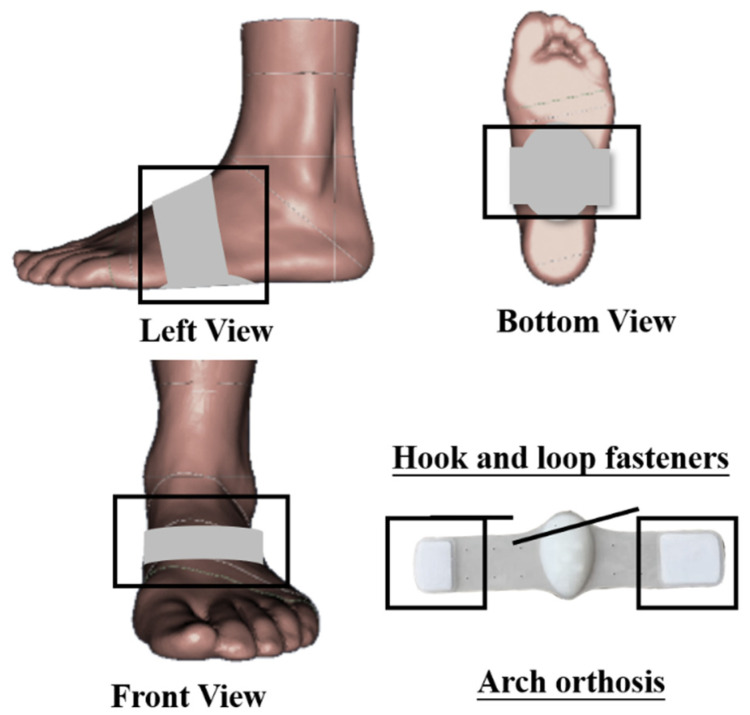
Illustration of arch support manipulation.

Once subjects completed static and dynamic arch evaluations (if the subject was determined to have flatfoot in all assessments), a 5 min warm-up and laboratory environment familiarization was conducted before gait task measurement. Tests of the AS manipulation with and without the AS orthoses were defined as AS and non-arch-support (NAS). Customized arch orthosis made of silicone material (peak longitudinal height of 20 mm) has extended hook and loop fasteners on both sides to attach barefoot (Figure 2). Each subject needed to walk barefoot along the walkway at self-selected speed again, under conditions of NAS and AS, respectively. Then, they were asked to stop walking quickly and safely when a termination signal was received, and their non-dominant leg stepped onto the ground and kept standing posture until they were instructed to continue walking. Detailed recording procedure of the kinematic data followed a previously established protocol [40]. The auditory signals were initiated using a bell, so hearing impairment should be excluded during the initial recruitment of subjects. A total of 20% of trials in each block involved the auditory signal, but the other 80% did not. There was a rest interval of 2 min between tests to minimize the influences of fatigue. Each subject was required to provide ten successful UGT trial datasets, including five trials under each condition.

A motion capture system (Vicon Motion System Ltd., Oxford, UK) containing eight infrared cameras was employed to capture the motion of lower-limb joints, with a frequency of 200 Hz. The joint kinematics was recorded from the dominant side in this experiment. Following a previously established protocol [40], fourteen reflective markers (9.0 mm) were placed on the surface of the subject to define the following anatomical segments: dominant shank (FLE: protrusion of the lateral epicondyle of the femur; FME: protrusion of the medial epicondyle of the femur; ST1-4: four shank tracking targets; LA: apex of the lateral malleolus; MA: apex of the medial malleolus), dominant forefoot (TOE: toe; D1M: 1st metatarsal, distal medial; D5M: fifth metatarsal, distal medial), and dominant hindfoot (HT1-3: heel tracking targets; LA; MA). All reflective markers were secured with skin-medical tape to avoid drop-off while moving. The MPJ was recognized as the angle between the forefoot and hindfoot anatomical coordinate systems, referring to the previous definition approach [41].

### 2.3. Data Collection and Processing

#### 2.3.1. Inverse Kinematics

For each gait trial, Visual 3D software (C-Motion Inc., Germantown, MD, USA) was employed to calculate the MPJ and ankle joint parameters in the sagittal, frontal, and transverse planes based on the C3D format files created by Vicon Nexus software (Vicon Motion System Ltd., Oxford, UK). A second-order low-pass Butterworth filter with a cutoff frequency of 6 Hz was adopted to denoise the marked trajectory [40]. An inverse kinematics algorithm was executed in Visual 3D software to calculate MPJ and ankle angles during the stance phase of UGT. For each UGT trial, joint angles were adjusted based on the stance phase and standardized to 101 time points.

#### 2.3.2. Coordination and Coordination Variability

As a non-linear method, the vector coding analysis technique quantifies the coordination pattern by inferring coupling angle (CA) in the angle-angle diagram to avoid miss segmental spatial data of motion [42]. This study used an optimized vector coding technique to calculate inter-joint coordination and CV, following the procedure reported by Needham et al. [43]. The CA ($\gamma_{i}$) was computed according to the successive proximal segment angles (ankle angle, $\theta_{A(i)}$) and distal segments angles (MPJ angle, $\theta_{M(i)}$). Mathematically, $\gamma_{i}\;$was inferred as:

(1) $$\gamma_{i}=\{\begin{array}{c} \tan^{-1}\left(\frac{\theta_{M\left(i+1\right)}{-\theta }_{M\left(i\right)}}{\theta_{A\left(i+1\right)}{-\theta }_{A\left(i\right)}}\right)\cdot \frac{180{}^{\circ}}{\pi }{,\;\mathrm{if}\;\theta }_{A\left(i+1\right)}{-\theta }_{A\left(i\right)}>0{}^{\circ}\\ \tan^{-1}\left(\frac{\theta_{M\left(i+1\right)}{-\theta }_{M\left(i\right)}}{\theta_{A\left(i+1\right)}{-\theta }_{A\left(i\right)}}\right)\cdot \frac{180{}^{\circ}}{\pi }{+180{}^{\circ},\;\mathrm{if}\;\theta }_{A\left(i+1\right)}{-\theta }_{A\left(i\right)}<0{}^{\circ} \end{array}$$

where i is the corresponding time point of standardized stance phase during UGT (i_1_ = the first stride in 101 time points, … i_n_ = the nth stride in 101 time points).

The following supplementary conditions were adopted [43]:

(2) $$\gamma_{i}=\{\begin{array}{c} 90{}^{\circ},\;\mathrm{if}\;\theta_{A(i+1)}{-\theta }_{A(i)}{=0{}^{\circ}\;\mathrm{and}\;\theta }_{M(i+1)}{-\theta }_{M(i)}>0{}^{\circ}\\ {-90{}^{\circ},\;\mathrm{if}\;\theta }_{A(i+1)}{-\theta }_{A(i)}{=0{}^{\circ}\;\mathrm{and}\;\theta }_{M(i+1)}{-\theta }_{M(i)}<0{}^{\circ}\\ {-180{}^{\circ},\;\mathrm{if}\;\theta }_{A(i+1)}{-\theta }_{A(i)}{<0{}^{\circ}\;\mathrm{and}\;\theta }_{M(i+1)}{-\theta }_{M(i)}=0{}^{\circ}\\ {\mathrm{undefined},\;\mathrm{if}\;\theta }_{A(i+1)}{-\theta }_{A(i)}{=0{}^{\circ}\;\mathrm{and}\;\theta }_{M(i+1)}{-\theta }_{M(i)}=0{}^{\circ} \end{array}$$

Then $\gamma_{i}$ for two joints was further adjusted in the range of 0°–360° based on the following equation [43,44,45]:

(3) $$\gamma_{i}=\{\begin{array}{c} \gamma_{i}{+360{}^{\circ},\;\mathrm{if}\;\gamma }_{i}<0{}^{\circ}\\ \gamma_{i}{,\;\mathrm{if}\;\gamma }_{i}\geq 0{}^{\circ} \end{array}$$

**Figure 3:**
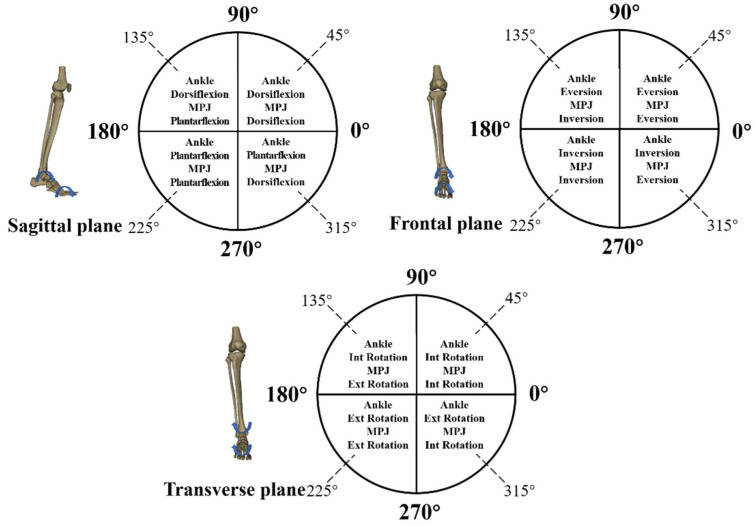
Classification of coordination model based on the coupling angle in sagittal, frontal, and transverse planes.

Selected segment couplings included ankle sagittal plane vs. MPJ sagittal plane, ankle frontal plane vs. MPJ frontal plane, and ankle transverse plane vs. MPJ transverse plane. Figure 3 illustrates the classification of coordination patterns from the CA in sagittal, frontal, and transverse planes [28].

Given the directivity of the CA, the average CA ($\bar{\gamma_{i}}$) were calculated by circular statistics [43,46,47], where $\bar{x_{i}}$ and $\bar{y_{i}}$ denotes the horizontal and vertical elements of the$\;\bar{\gamma_{i}}$ at per time point (i) of the stance phase during UGT. Then $\bar{\gamma_{i}}$ for two joints was further adjusted in the range of 0–360° according to Equation (5).

(4) $$\bar{x_{i}}=\frac{1}{n}\overset{n}{\underset{i=1}{\sum }}{\cos\gamma }_{i};\;\bar{y_{i}}=\frac{1}{n}\overset{n}{\underset{i=1}{\sum }}{\sin\gamma }_{i}$$

(5) $$\bar{\gamma_{i}}=\{\begin{array}{c} \tan^{-1}(\frac{\bar{y_{i}}}{\bar{x_{i}}})\cdot \frac{180{}^{\circ}}{\pi }{,\;\mathrm{if}\;x}_{i}{>0{}^{\circ}\;\mathrm{and}\;y}_{i}>0{}^{\circ}\\ \tan^{-1}(\frac{\bar{y_{i}}}{\bar{x_{i}}})\cdot \frac{180{}^{\circ}}{\pi }{+180{}^{\circ},\;\mathrm{if}\;x}_{i}<0{}^{\circ}\\ \tan^{-1}(\frac{\bar{y_{i}}}{\bar{x_{i}}})\cdot \frac{180{}^{\circ}}{\pi }{+360{}^{\circ},\;\mathrm{if}\;x}_{i}{>0{}^{\circ}\;\mathrm{and}\;y}_{i}<0{}^{\circ}\\ {90{}^{\circ},\;\mathrm{if}\;x}_{i}{=0{}^{\circ}\;\mathrm{and}\;y}_{i}>0{}^{\circ}\\ {-90{}^{\circ},\;\mathrm{if}\;x}_{i}{=0{}^{\circ}\;\mathrm{and}\;y}_{i}<0{}^{\circ}\\ {\mathrm{undefined},\;\mathrm{if}\;x}_{i}{=0{}^{\circ}\;\mathrm{and}\;y}_{i}=0{}^{\circ} \end{array}$$

Lastly, the equation below was used to infer the coupling angle variability (CAV, ${\mathrm{CAV}}_{i}$):

(6) $${\mathrm{CAV}}_{i}=\sqrt{2\cdot \left(1-\bar{r_{i}}\right)}\cdot \frac{180{}^{\circ}}{\pi }\;,\;\mathrm{where}\;\bar{r_{i}}=\sqrt{{\bar{x_{i}}}^{2}+{\bar{y_{i}}}^{2}}$$

where $\bar{r_{i}}$ is the length of average coupling angle per time point (i) of the standardized stance phase during UGT.

### 2.4. Statistical Analysis

In this study, statistical analysis was applied to explore the differences between results under conditions of NAS and AS. A paired-sample *t*-test was applied in SPSS version 23.0 software (Chicago, IL, USA) to compare discrete MPJ and ankle angle, coordination, and CV in every sub-phase of UGT. The stance phase of UGT was divided into the following three gait sub-phases: stimulus delay (SD, 0~38% of stance), reaction time (RT, 39~65% of stance), and residual stance time (RST, 66~100% of stance) [20,43]. ES using Cohen’s d for each significant result were measured in the GPower software, and ES was divided into three levels: small (0.2 < ES < 0.5), medium (0.5 ≤ ES < 0.8), and large (ES ≥ 0.8) [44].

For time-varying MPJ and ankle joint angle as well as mean CA, normality was examined before statistical analysis adopting a paired-sample *t*-test in the one-dimensional statistical parametric mapping (SPM). Because of the one-dimensional time series characteristics, the open-source SPM 1d package, which verifies data variability according to random vector field theory, was used for the statistical analysis [45,46]. The open-source script of SPM 1d (paired-samples *t*-test) was used in MATLAB R2016a (The MathWorks, Natick, MA, USA). A significance level of 5% was set for all statistical analyses.

## 3. Results

### 3.1. Joint Kinematics

**Figure 4:**
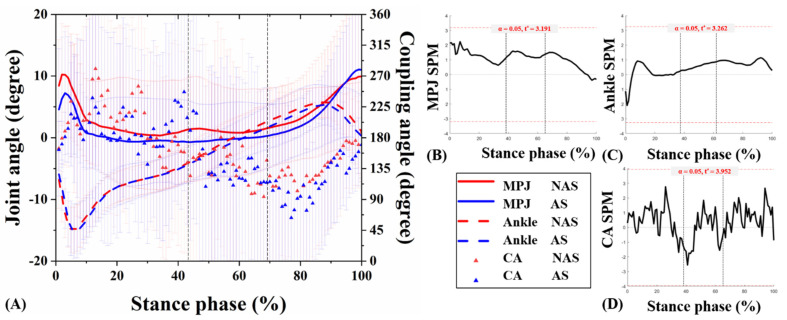
Angle curves of the ankle and MPJ, and mean CA for MPJ-ankle in the sagittal plane (**A**). The SPM results between NAS and AS during UGT, depicting the MPJ angle (**B**), ankle angle (**C**), and CA (**D**). MPJ: metatarsophalangeal joint; NAS: non-arch-support; AS: arch-support; CA: coupling angle; SPM: statistical parametric mapping; UGT: unplanned gait termination.

**Figure 5:**
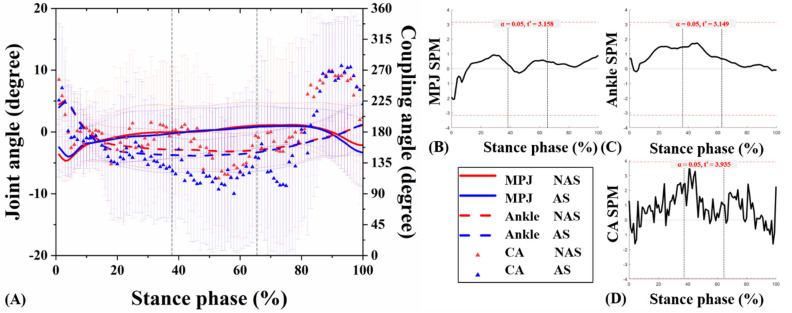
Angles curves of the ankle and MPJ, and mean CA for MPJ-ankle in the frontal plane (**A**). The SPM results between NAS and AS during UGT, depicting the MPJ angle (**B**), ankle angle (**C**), and CA (**D**).

**Figure 6:**
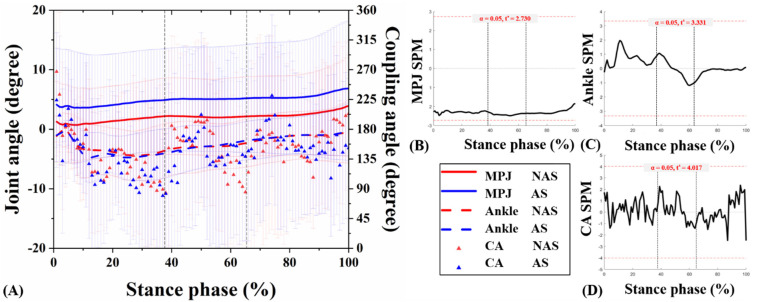
Angles curves of the ankle and MPJ, and mean CA for MPJ-ankle in the transverse plane (**A**). The SPM results between NAS and AS during UGT, depicting the MPJ angle (**B**), ankle angle (**C**), and CA (**D**).

The joint kinematic patterns of the ankle and MPJ in three motion planes were plotted against the percentage of stance for the UGT from subjects under NAS and AS conditions (Figure 4A, Figure 5A, Figure 6A). The detailed mean joint angles in each sub-phase of UGT are shown in Table 1.

Significant results related to MPJ kinematics appeared only in the transverse plane. Specifically, during RT of UGT, the mean MPJ angle of NAS was −0.89 ± 0.41°, and the angle of AS was −0.34 ± 0.68° (*p* = 0.026). Compared with NAS, AS presented an external rotation decrease in the transverse plane during RST of UGT (*p* = 0.024). No significant difference was observed in the ankle joint angle between NAS and AS in three planes during sub-phases of UGT (all *p* > 0.05). Besides, the SPM1D test exhibited no statistically significant difference between the ankle and MPJ angles obtained from the subjects under NAS and AS sessions during UGT (*p* > 0.05) (Figure 4B, Figure 5B, Figure 6B, and Figure 4C, Figure 5C, Figure 6C, respectively).

### 3.2. MPJ-Ankle Coordination Pattern

CA curves of the ankle and MPJ in the three motion planes were plotted against the percentage of stance phase for the UGT from subjects under NAS and AS conditions (Figure 4A, Figure 5A, Figure 6A). Table 1 compares the mean CA between two conditions during each sub-phase of UGT. Nevertheless, according to the SPM1D analysis, no statistical difference was recognized under NAS and AS conditions during UGT (Figure 4D, Figure 5D, Figure 6D).

In the sagittal plane during SD, the mean CA under AS condition was 187.59 ± 44.89° (changed from 207.85 ± 32.52° under NAS condition), which was close to 180°, indicating an MPJ-dominant coordination strategy. However, it was observed that each sub-phase exhibits no significant difference between the two conditions (all *p* > 0.05).

In the frontal plane during SD, the mean CA under the NAS condition was 177.16 ± 27.41°. It was 157.75 ± 32.54° under AS condition (*p* = 0.026), indicating that the subjects moved the MPJ into inversion and ankle into eversion. At the same time, they had more ankle motion compared with MPJ motion after supporting the arch. Furthermore, during RT, the mean CA under NAS condition was 147.96 ± 67.48°, and it was 116.78 ± 55.91° under AS condition, which indicates that even though subjects experienced MPJ inversion and ankle eversion, they showed more ankle motion under AS compared to NAS. Meanwhile, no significant difference was found under these two conditions (*p* > 0.05).

In the transverse plane during SD, though the subjects experienced MPJ external rotation and ankle internal rotation under NAS (137.49 ± 30.22°) and AS (130.83 ± 30.98°), they revealed a more anti-phase pattern under NAS. Similarly, during RT and RST, subjects moved the MPJ into the external rotation while moving the ankle into internal rotation; they had more ankle motion than MPJ motion under AS conditions.

### 3.3. MPJ-Ankle Coordination Variability

Table 1 shows CAV values under NAS and AS conditions per sub-phase of UGT; both exhibit no statistical difference under the two conditions (all *p* > 0.05).

In the sagittal plane sub-phases of UGT, compared with NAS, the CAV of subjects under AS condition increased slightly, from 64.05–76.70° to 69.88–77.21°. On the contrary, in the frontal plane during SD and RST, slight decreases were found in CAV after supporting patients’ arch; nevertheless, there was no significant difference between before and after. Likewise, a similar trend was exhibited in the transverse plane, that is, the slight decrease during SD and RST as well as the increase during RT in the AS session.

## 4. Discussion

The significance of a well-functioning arch has long been recognized when performing daily gait tasks. The fundamental purpose of this study was to explore foot–ankle coordination and CV in patients with flatfoot of AS manipulation during UGT. Manipulation in the foot arch might potentially influence patients’ MPJ–ankle coordination patterns when facing unexcepted stimulus. Meanwhile, present data showed no significant difference caused by acute AS in the MPJ-ankle CV in sagittal, frontal, and transverse planes during sub-phases of UGT.

The most anatomically essential characteristic of flatfoot is the medial longitudinal arch’s partial or complete loss (collapse), with clinical impact ranging from slight impediment to intense pain leading to life disorders [48]. The longitudinal AS mechanism formed by the foot orthosis mainly relies on transferring the regional impulse to the medial foot to resist the depression of the arch. Because the plantar aponeurosis is under tension when loading the foot, strain can be reduced through proper arch control manipulation [49]. A previous study [50] found that a 3-mm AS could stimulate the arch, causing an immediate rising in ipsilateral dynamic plantar loading and a contralateral displacement of the center of pressure in bilateral standing. The joint kinematics data in this study further supports the above results; the subjects’ ankle joints show a greater tendency to inversion under AS conditions. Meanwhile, a significant difference was exhibited in the MPJ transverse plane during RT and RST of UGT (*p* = 0.04–0.026). This abating tendency of MPJ to external rotation caused by acute AS might associate with compensatory muscle activation strategies to modify the posture and maintain foot stability [50]. In addition, Nakajima et al. [51] and Hatfield et al. [52] found that AS insoles can effectively reduce knee adduction moment during walking by 13.3% and 6%, respectively. When the subjects received an unplanned signal, their calf muscles, such as gastrocnemius and soleus, contracted to stimulate the ankle joint [50,53]. Janin and Dupui [50] used the position control of the central nervous system to explain the biomechanical changes of the lower limbs caused by the AS, including plantar pressure distribution. To be specific, the central nervous system may interpret the sensory stimulation caused by the arch manipulation as a disturbance of the COM toward the stimulus source and then may respond with compensatory strategies of muscle activity to adjust the lower limbs kinematics represented by the MPJ and ankle joints to stay away from the stimulation [50].

For results of MPJ–ankle coordination pattern, frontal plane MPJ-ankle coordination under AS during stimulus delay significantly decreased from 177.16 ± 27.41° to 157.75 ± 32.54° compared with under NAS (*p* = 0.026), which means the patients moved the MPJ into inversion and moved the ankle into eversion, respectively. In addition, the data revealed a greater range of ankle movement than MPJ after supporting the arch. A study by Jafarnezhadgero et al. [28] found that after four months of AS manipulation intervention, the patients with flexible flatfoot displayed a lower-extremity inter-joint coordination mode indicating more inversion motion in the ankle during mid-stance than before the intervention. However, they explored the effects of wearing foot orthoses for a long time while using only the most conventional gait tests; hence it is not appropriate to compare previous findings with the results of our experiment. AS manipulation is naturally made to adjust hindfoot eversion, although different AS heights may have weak influences of eversion shifting and the limitations of tibia intorsion [54]. The proportion of ankle eversion would be adjusted under AS conditions to improve inter-joint relative coordination. Moreover, Deborah et al. [55] reported a correction effect for rotations which occurs from the initial contact phase to peak internal rotation of the tibia during running and the coordination relationship between the calcaneal inversion/eversion and tibial axial rotation. Since the initial stance phase of gait termination and running have similar biomechanical mechanisms [22], our results confirmed the abovementioned issue, which displayed more ankle movement than MPJ in RT and RST under AS condition. This might improve the shock absorption, reduce the load accumulated on the foot, and achieve the positive effect of relieving joint pain and improving stability after orthopedic intervention based on the change of the inter-joint CA [28,56,57,58].

Considering that a previous study [59] found that shoe insole had no impact on the variability of lower limb joints couplings during landing, this study hypothesized that acute AS would not significantly affect inter-joint CV in patients with mild flatfoot. This study also verified the above hypothesis that there was no significant difference caused by acute AS in the MPJ-Ankle CV in sagittal, frontal, and transverse planes during sub-phases of UGT. A previous study [60] reported that customized foot orthoses might be crucial in maintaining the coordinated variability between ankle inversion and eversion and knee internal rotation and external rotation. Meanwhile, Jafarnezhadgero et al. [28] also found decreased CV during push-off after 16-week wearing AS orthoses. Nevertheless, in this study about acute arch support manipulation intervention, although a slight reduction in MPJ-ankle CV values in the transverse plane was observed during SD and RST of UGT, the difference was not significant, which may be because the immediate effects from the arch manipulation intervention were not enough to impact MPJ-ankle CV potentially. Most studies [14,37,49,50,51,52,54,58] only quantify the instantaneous or short-term impact of wearing a foot orthosis on biomechanical variables. Still, the foot orthosis has been clinically used for a long time, so it may take a while for patients to adapt [28,61,62,63,64]. This study took CV to quantify the fluctuations in the timing and magnitude of relative motions of MPJ and ankle joints during UGT. Future studies could be further applied to the long-term arch manipulation intervention to reflect the biomechanical effects of AS on lower-extremity inter-segment coordination and variability linked to foot injury susceptibility during gait termination.

However, several limitations should be considered. First, all participants were young males, which resulted from the motivation to alleviate gender- and age-related differences of locomotion function. Second, the subjects in this study were required to perform UGT tasks barefoot in a laboratory environment, which is different from where gait termination occurs in real life. The original intention of this design is to show the essential foot kinematic characteristics. Ultimately, the present study only focused on the acute effects of arch manipulation. Nevertheless, long-term follow-up studies are also worth exploring, revealing further arch knowledge associated with related injury susceptibility.

## 5. Conclusions

The present experiment conducted a prospective exploration into the immediate effects of AS intervention on foot-ankle coordination and CV alterations. In addition, these results might help understand the implications of manipulating foot or arch structure on foot injury during UGT. Related information might be necessary for further considering the utility of intrinsic foot manipulations such as foot surgical intervention and extrinsic foot manipulations such as designing specific shoes.

## Figures and Tables

**Table 1:** The joint angle of MPJ and ankle, mean coupling angle, and coupling angle variability [mean (standard deviation)] per sub-phase of unplanned gait termination.

Plane	Sub-Phase	Mean Joint Angle				Mean Coupling Angle		Coupling Angle Variability	
		MPJ		Ankle					
		NAS vs. AS	Mean Difference (95% CI), ES	NAS vs. AS	Mean Difference (95% CI), ES	NAS vs. AS	Mean Difference (95% CI), ES	NAS vs. AS	Mean Difference (95% CI), ES
Sagittal	SD	1.98 (6.96) −0.18 (4.15)	1.14 (−0.21–4.52)	−9.03 (2.44) −9.04 (2.74)	0.58 (−1.18–1.20)	207.85 (32.52) 187.59 (44.89)	10.10 (−0.64–41.15)	64.05 (4.73) 69.88 (3.45)	1.03 (−2.97–1.30)
	RT	2.66 (6.61) 1.07 (4.84)	1.20 (−0.90–4.07)	−1.60 (3.03) −2.61 (2.19)	0.81 (−0.66–2.68)	135.60 (71.86) 138.08 (38.64)	16.29 (−36.58–31.61)	75.60 (2.29) 75.84 (2.36)	0.71 (−1.70–1.21)
	RST	4.95 (8.01) 4.25 (5.85)	1.64 (−2.69–4.08)	3.97 (2.27) 3.18 (2.80)	0.58 (−0.43–1.99)	110.89 (39.23) 102.25 (44.32)	10.59 (−13.52–30.81)	76.70 (1.69) 77.21 (1.91)	0.53 (−1.61–0.59)
Frontal	SD	−2.92 (2.05) −2.74 (1.08)	0.39 (−1.01–0.64)	−0.87 (2.57) −1.69 (1.45)	0.65 (−0.53–2.18)	177.16 (27.41) 157.75 (32.54)	8.15 (2.59–36.23) 0.48 *	69.39 (5.52) 69.25 (4.80)	1.19 (−2.32–2.60)
	RT	−1.91 (1.37) −1.42 (0.91)	0.34 (−1.22–0.24)	−3.02 (2.91) −3.40 (2.70)	0.55 (−0.45–1.80)	147.96 (67.48) 116.78 (55.91)	15.81 (−1.45–63.81)	75.89 (2.31) 76.08 (1.42)	0.65 (−1.55–1.16)
	RST	0.19 (4.26) −0.20 (3.58)	0.81 (−1.29–2.06)	−1.22 (2.98) −1.34 (2.84)	0.49 (−0.88–1.13)	210.00 (38.02) 192.34 (51.74)	16.19 (−15.77–51.07)	76.40 (1.77) 77.07 (1.70)	0.44 (−1.58–0.24)
Transverse	SD	−1.74 (0.33) 1.47 (0.81)	0.24 (−1.00–0.00)	−3.11 (3.82) −3.49 (3.04)	0.50 (−0.65–1.43)	137.49 (30.22) 130.83 30.98)	6.77 (−7.30–20.63)	71.26 (2.63) 71.08 (2.85)	1.00 (−1.90–2.25)
	RT	−0.89 (0.41) −0.34 (0.68)	0.22 (−1.02–−0.07) 0.61 *	−2.34 (3.67) −2.31 (2.99)	0.43 (−0.93–0.86)	151.26 (41.19) 144.80 (57.87)	12.83 (−20.02–32.95)	76.37 (2.02) 75.78 (2.51)	0.68 (−0.81–1.99)
	RST	−1.02 (0.43) −0.36 (0.90)	0.26 (−1.20–−0.10) 0.63 *	−1.19 (4.09) −1.12 (3.27)	0.43 (−0.96–0.83)	163.70 (42.07) 155.80 (58.33)	11.89 (−16.76–32.57)	76.74 (1.69) 76.51 (2.50)	0.56 (−0.93–1.40)

Note: Unit for the values is degrees (°); MPJ: metatarsophalangeal joint; NAS: non-arch-support; AS: arch-support; CI: confidence interval; ES: effect size; The sub-phases are categorized into three events: stimulus delay (SD, 0~38% of stance), reaction time (RT, 39~65% of stance), and residual stance time (RST, 66~100% of stance); * significance difference between NAS and AS, with *p* < 0.05 (bolded values).

## Data Availability

The data presented in this study are available on request from the corresponding author. The data are not publicly available due to ethical considerations.
